# Global, regional, and national burden of hypertensive heart disease during 1990–2019: an analysis of the global burden of disease study 2019

**DOI:** 10.1186/s12889-022-13271-0

**Published:** 2022-04-27

**Authors:** Yunyan Lu, Tian Lan

**Affiliations:** 1 Department of Cardiology, The First People’s Hospital of Xiaoshan District, Xiaoshan Affiliated Hospital of Wenzhou Medical University, Hangzhou, Zhejiang People’s Republic of China; 2 Department of Breast Surgery, Hangzhou TCM Hospital Affiliated to Zhejiang Chinese Medical University, Hangzhou Hospital of Traditional Chinese Medicine, Hangzhou, Zhejiang People’s Republic of China

**Keywords:** Hypertensive heart disease, Global disease burden, Time trends, Socioeconomic factors

## Abstract

**Background:**

Hypertensive heart disease (HHD) is a major public health issue worldwide. We analyzed the global, regional, and national burden of HHD between the years 1990 and 2019 in relation to age, gender, and socioeconomic factors.

**Methods:**

The prevalence and death rates, the disability adjusted life-years (DALY), and the corresponding age-standardized rates of HHD were extracted from the Global Burden of Disease study 2019. The epidemiological trends were evaluated by calculating the estimated annual percentage changes (EAPC) of the above variates.

**Results:**

A total of 19.60 million HHD cases were documented in 2019 compared to 7.82 million in 1990, corresponding to an EAPC of 0.17. Contrarily, the global age-standardized death rate (ASDR) and age-standardized DALYs decreased with respective EAPCs of − 0.74 and − 1.02. HHD mostly occurred in people aged over 65. The disease burden of HHD varied considerably between countries, and univariate linear regression indicated that many socioeconomic variables had significantly negative correlations with age-standardized DALY rate.

**Conclusion:**

HHD cases have increased over the last three decades; however the mortality rate has declined. Multi-faceted improvements in health, education and income could help to alleviate the disease burden of HHD, specially in some regions with lower socio-demographic index and higher ASDR.

**Supplementary Information:**

The online version contains supplementary material available at 10.1186/s12889-022-13271-0.

## Background

Hypertensive heart disease (HHD), which includes left ventricular hypertrophy, systolic and diastolic dysfunction, and a broader spectrum of cardiac and vascular adaptations, is a major public health issue worldwide that generates complications and damages quality of life [1–3]. From 1990 to 2017, the age-standardized prevalence rate (ASPR) per 100,000 of HHD grew to 202.8 by 7.3%. While, the age-standardized death rate (ASDR) decreased from 15.22 to 12.28 in the same period [4]. In the 2017 American College of Cardiology and American Heart Association Guidelines, hypertension has been redefined as a systolic blood pressure of ≥130 mmHg and/or a diastolic blood pressure of ≥80 mm [5]. The shift of hypertension definition led to an increase in hypertension prevalence from 18.9 to 43.5% [6], which may augment the reported HHD burden.

Although it was reported that the burden of HHD has increased significantly in Iran and Poland [4, 7], there have been few studies focused on the global burden of HHD [8]. No comprehensive update of the descriptive epidemiology and trends of HHD has been released since the Global Burden of Disease, Injuries, and Risk Study (GBD) 2017. The GBD 2019 provides an independent estimation of prevalence, mortality, disability-adjusted life-years (DALYs) due to HHD with its updated population-based data and methodological refinements, for two sexes in 204 countries and territories, which presented us an opportunity to explore the global disease burden of HHD [9].

In this study, we provided the HHD burdens (prevalence, death, DALYs, and corresponding age-standardized rate [ASR]) according to age, gender, and geographical location, and assessed their relationships to socioeconomic factors. Our findings may help the policymakers to understand comprehensively the global burden of this disease and allocate reasonably the limited health resources, such as human resources and health spending, for closing the gaps in the global HHD disparities.

## Methods

### Study data sources

This was a secondary analysis based on the GBD 2019, which summarized the global burden of 354 diseases and injuries in 204 countries and territories between 1990 and 2019 [9]. The estimates and their 95% uncertainty intervals (UIs) for prevalence, death, DALY, and the respective ASRs of HHD were extracted online via the Global Health Data Exchange (GHDx) query Tool (http://ghdx.healthdata.org/gbd-results-tool). This study received exemption from the Ethics Committee of Hangzhou Hospital of Traditional Chinese Medicine as no patients, physicians, or hospital identifiers were examined.

To investigate the correlations between the socioeconomic status of countries and the country-level disease burden of HHD, we extracted the socio-demographic index (SDI), human development index (HDI), inequality-adjusted human development index (IHDI), health access and quality (HAQ), proportion of people receiving at least some secondary education, and number of physicians per 10,000 people from public databases. The SDI is a composite index reflecting a country’s socio-demographic development status. And it was collected and divided into five categories (low, low-middle, middle, high-middle, and high SDI) in the GBD 2019 (http://ghdx.healthdata.org/sites/default/files/record-attached-files/IHME_GBD_2019_SDI_1990_2019_Y2020M10D15.XLSX). The HDI extracted from the United Nations Human Development Report (http://hdr.undp.org/en/data) is an important measurement of achievement in three dimensions of human development across countries, such as gross national income, life expectancy, and education. Additionally, the IHDI, a distribution-sensitive average value of human development, was download from the United Nations Human Development Report (http://hdr.undp.org/en/data). The HAQ is a single and interpretable summary value from the GHDx (http://ghdx.healthdata.org), providing a strong basis for progress toward greater availability and higher-quality personal health care [10]. The percentage of the population over 25 that has reached secondary level education and the number of physicians per 10,000 people, which reflect a national educational level and health care infrastructure, respectively, were also downloaded from the United Nations Human Development Report.

### Statistical analysis

We used the ASRs of prevalence, death, and DALY and the corresponding estimated annual percentage changes (EAPC) to quantify temporal trends in the disease burden of HHD. DALYs were calculated as the sum of years lived with disability due to HHD and the years of life lost due to premature death [11]. ASRs are often used to make such comparisons between two time periods or two different geographical areas, as we take into consideration the differences in the age structure of the populations being compared. The EAPC is a common and significant evaluation of the ASR changing trend during a specified interval [12, 13]. The natural logarithm of ASR along with year was controlled by a relevant linear regression mathematical model.

$$y=\mathrm{a}+\upbeta \mathrm{x}+\upvarepsilon$$

y referred to ln(ASR), x represented calendar year, and ɛ referred to error term.

Subsequently, the EAPC was estimated as 100 × (exp(β) − 1) and 95% confidence interval (CI) [14, 15]. It presented an upward trend of the ASR when the upper and lower boundaries of EAPC were both positive. On the contrary, it was a backward trend of the ASR when the upper and lower boundaries of EAPC were both negative. Otherwise, the ASR was considered as a stable status [16]. Univariate linear regression analysis was performed to access the relationship between the socioeconomic status of countries and the country-level disease burden of HHD. Pearson’s correlation analysis was conducted to explore the relationship between the ASR in 2019 and the corresponding SDI. All methods were performed using relevant guidelines. All analyses were performed with R software (version 3.5.2). We cleaned the data with the R packages “tidyverse” and “stringr”, and the R package “ggplot2” was used for data visualization. *P* value less than 0.05 was set as a significant difference.

## Results

### The change in the prevalence of HHD

**Fig. 1:**
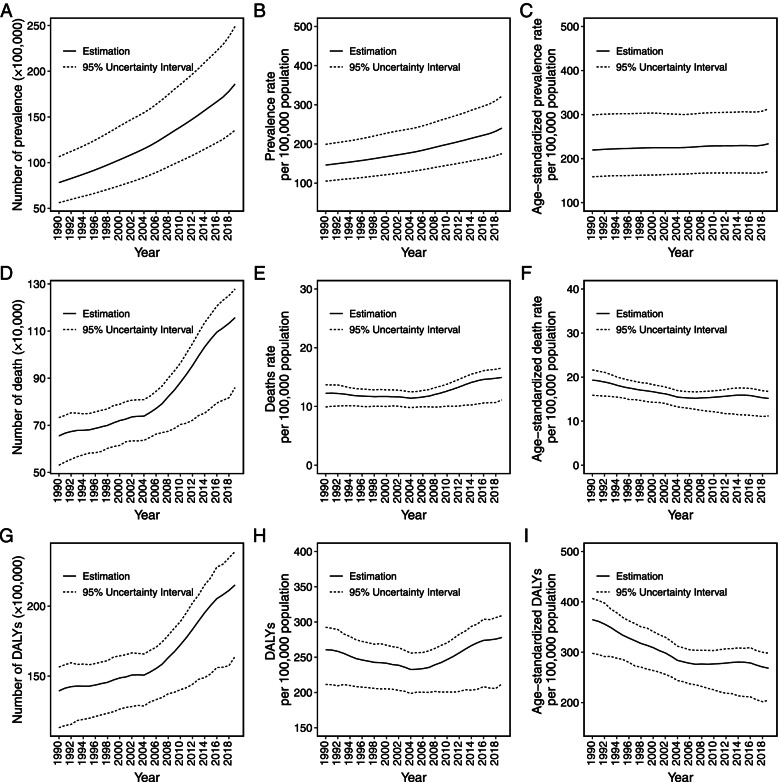
The global trend of hypertensive heart disease from 1990 to 2019. The number of prevalence (**A**), death (**D**), and DALY (**G**). The rate of prevalence (**B**), death (**E**), and DALY (**H**). Age-standardized rate of prevalence (**C**), death (**F**), and DALY (**I**). Dashed lines represent 95% uncertainty interval; DALY, disability adjusted life-year

At the global level, the prevalence of HHD increased by 137.91% from 7.82 million in 1990 to 19.60 million in 2019 (Fig. 1A, Table S1). The prevalence rate went up year by year, while the ASPR was relatively stable (Fig. 1C). The ASPR was 233.77 (95% UI = 170.52–312.9) per 100,000 population in 2019, which increased slightly compared with that in 1990 with an EAPC of 0.17 (95% UI = 0.15–0.18) (Fig. 1C, Tables S2 and S3). Compared with the ASPR trend of the female subjects (EAPC, 0.28, 95% UI = 0.26–0.30), the trend of the male subjects was more stable during the study period (EAPC, 0.02, 95% UI = 0.00–0.04, Table S3).

**Fig. 2:**
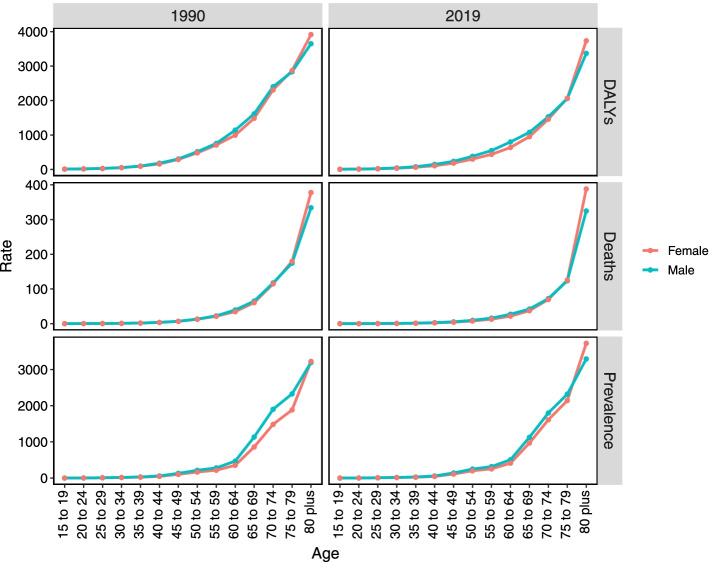
The gender-specific global prevalence, death, and DALY rate of hypertensive heart disease in 1990 and 2019. The vertical axis represents DALY, death, and prevalence rate (per 100,000 population). DALY, disability adjusted life-year

HHD occurred mostly in people aged over 65 (Fig. S1A). We also found that the ASPR increased with age growth for both men and women in 1990 and 2019. The female prevalence rate was much higher than male in people aged over 80 during 2019, yet there was a similar prevalence rate for aged men and women in 1990 (Fig. 2).

Among 25 GBD regions, top three regions with the highest prevalence cases were Asia, East Asia, and America. In addition, the three regions with the highest ASPR were East Asia (426.15, 95% UI = 306.64–574.76), Oceania (344.91, 95% UI = 248.54–477.87), and Southeast Asia (334.77, 95% UI = 244.81–451.58) (Table S4). At the national level, China carried the highest HHD prevalence, followed by the United States of America and India (Fig. S2A). The highest ASPR of HHD occurred in Cook Islands, Jordan, Kuwait and Seychelles (Fig. S2C).

### The change in the HHD mortality

A total of 1.16 (95% UI = 0.86–1.28) million people were estimated to experience HHD associated deaths worldwide in 2019, which increased from 0.65 (95% UI = 0.53–0.73) million death cases in 1990 (Table S1). The ASDR in females was 15.05 (95% UI = 11.51–17.09) per 100,000 population in 2019, which was moderately higher than that in males (14.95, 95% UI = 10.32–16.75) (Table S2). Although the number of HHD deaths grew up dramatically during 1990–2019, the trend of death rate was relatively stable and the global ASDR declined with a negative value of EAPC (− 0.74, 95% UI = -0.92--0.58) (Fig. 1D, E, and F, Table S3). Meanwhile, the male and female ASDR shared a similar trend (EAPC for men, − 0.72, 95% UI = -0.95--0.50; EAPC for women, − 0.79, 95% UI = -0.93--0.65).

For both men and women, age-specific distribution of death rate remained stable in 1990 and 2019 (Fig. 2). Like HHD prevalence, people aged over 65 were more likely to suffer HHD deaths (Fig. S1B).

At the regional level, Central Sub-Saharan Africa, Eastern Sub-Saharan Africa, North Africa and Middle East had the highest ASDR; Australasia, high-income Asia Pacific and Eastern Europe were the three regions with the lowest ASDR (Table S5). At the national level, China carried the highest HHD death burden, followed by India and the Untied States of America (Fig. S2D). Bulgaria, Afghanistan, and Central African Republic were the three countries with highest ASDR (Fig. S2F).

### The change in DALYs of HHD

A total of 21.50 (95% UI = 16.40–23.90) million DALYs were estimated on a global scale in 2019, and 13.94 (95% UI = 11.31–15.65) DALYs in 1990 (Table S1). There was a consistent rise in DALY number (Fig. 1G). However, DALY rate declined between 1990 and 2005, then ascended during 2006–2019 (Fig. 1H). In addition, it shown a persistent decline for the age-standardized DALY rate over the 30 years (Fig. 1I).

The age-standardized DALY rate in men was 277.86 (95% UI = 199.58–311.14) per 100,000 population in 2019, which was higher than that in women (256.81, 95% UI = 205.22–291.98) (Table S2). The DALY rate distribution for males and females in 2019 was similar to that in 1990 (Fig. 2). In 2019, the age-specific trends of DALY rate attributed to HHD were similar for both sexes.

**Fig. 3:**
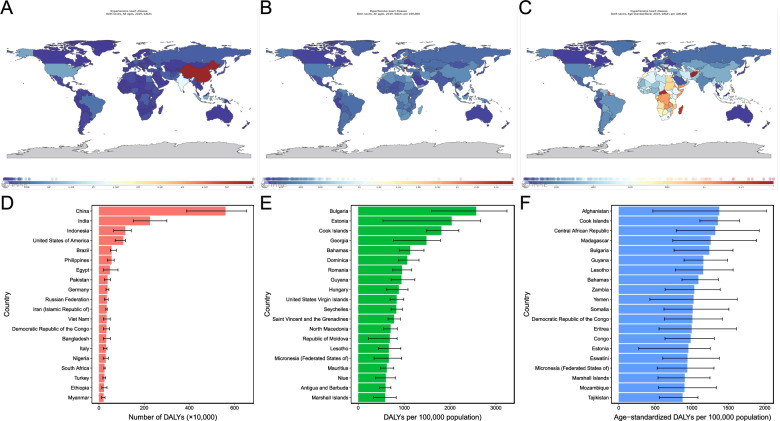
Global map of the disease burden of hypertensive heart disease (**A**, DALY number; **B**, DALY rates; **C**, Age-standardized DALY rates) and the top 20 countries with disease burden (**D**, DALY number; **E**, DALY rates; **F**, Age-standardized DALY rates)

On the observation of the regions scale, Central Sub-Saharan Africa, Eastern Sub-Saharan Africa, and Oceania were the three regions with the highest age-standardized DALY rates (Table S5). It revealed a considerable national disparity in the burden of HHD. DALY numbers varied more than 10-fold between countries (Fig. 3A). China had the highest HHD DALY number, followed by India and Indonesia (Fig. 3D). After adjusting population, Bulgaria, Estonia, and Cook Islands were the three countries with the highest rate of DALYs (Fig. 3B and E). After adjusting for age and population, Afghanistan, Cook Islands, and Central African Republic had the highest age-standardized DALY rates (Fig. 3C and F).

### The associations between the disease burden of HHD and sociodemographic factors

**Fig. 4:**
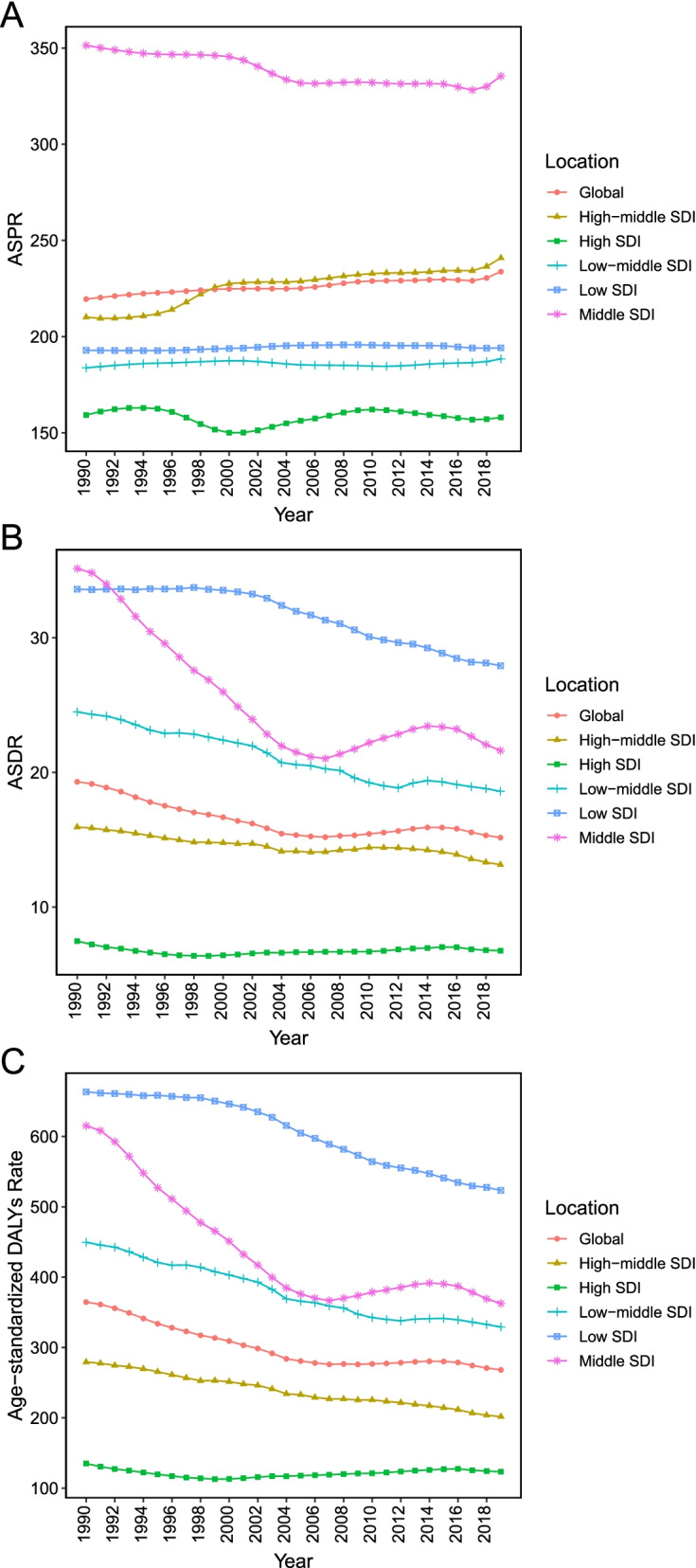
The age-standardized prevalence, death, and DALY rate for hypertensive heart disease by different SDI regions, 1990–2019. ASPR, age-standardized prevalence rate; ASDR, age-standardized death rate; DALY, disability adjusted life-year; SDI, socio-demographic index

The drift of HHD-related ASPR, ASDR, and age-standardized DALYs rate among five SDI quintiles were presented in Fig. 4. The ASPR of HHD was highest in the middle SDI region, and the lowest in the high SDI region between 1990 and 2019 (Fig. 4A). It was interesting to note that, as opposed to the regions with other SDI, the middle SDI region presented a descending trend of ASPR (EAPC, − 0.24, 95% UI = -0.2--0.20) (Table S3). ASDR and age-standardized DALY rate decreased the fastest in the middle SDI region (EAPC, − 1.58, 95% UI = -1.98--1.20 for ASDR; EAPC, − 1.74, 95% UI = -2.11--1.41 for age-standardized DALY rate) (Table S3, Fig. 4B and C). In the middle SDI region, the trend of ASDR and age-standardized DALY rate presented undulating curves (Fig. 4B and C). Compared with a downward trend for females (EAPC, − 0.28, 95% UI = -0.4--0.11), male age-standardized DALY rate showed an upward tendency in the high SDI region (EAPC, 0.34, 95% UI = 0.11–0.57).

**Fig. 5:**
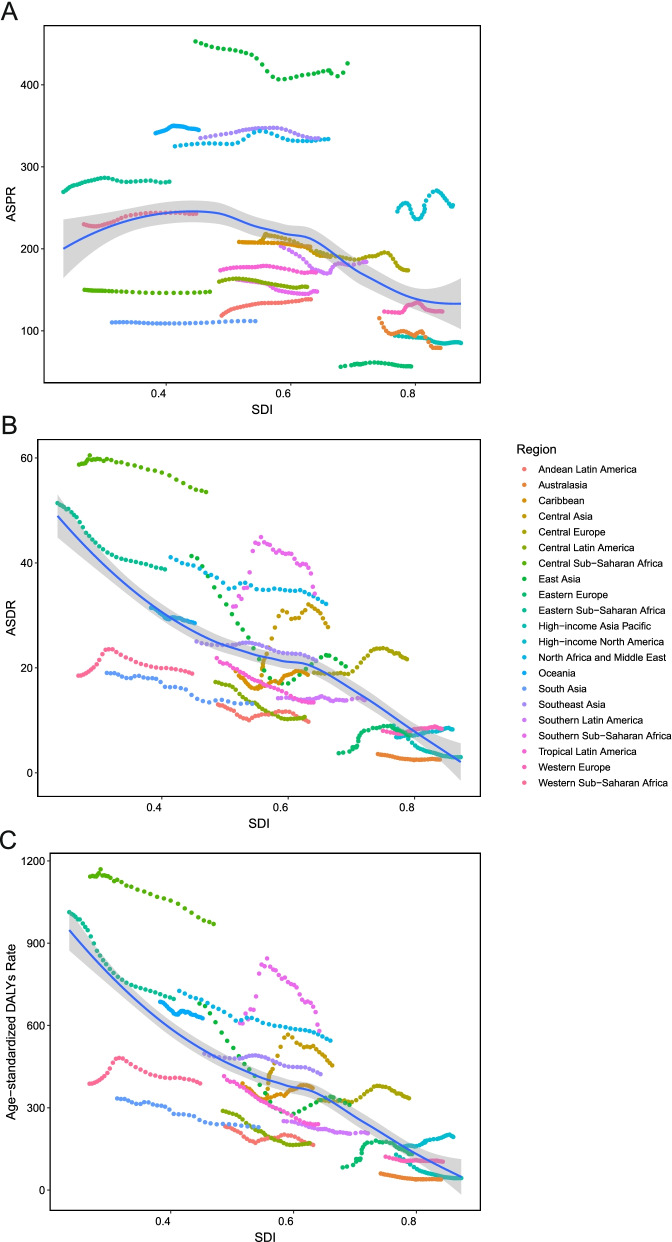
The trend in ASPR (**A**), ASDR (**B**), age-standardized DALY rate (**C**) of hypertensive heart disease in 21 regions based on SDI. Expected values are shown as the dark blue line. ASPR, age-standardized prevalence rate; ASDR, age-standardized death rate; DALY, disability adjusted life-year; SDI, socio-demographic index

ASPR, ASDR, and age-standardized DALY rate of HHD stratified by SDI were shown in Fig. 5. ASPR of HHD rose before SDI value of 0.4 and then start to decrease (Fig. 5A). There was a negative and significant Pearson’s correlation between HHD disease burden and SDI (*r* = − 0.74, 95% CI = -0.77--0.70, *p* < 0.001, for age-standardized DALY rate; *r* = − 0.70, 95% CI = -0.74--0.66, *p* < 0.001, for ASDR) (Fig. 5C). The univariate linear regression indicated that many socioeconomic variables (HDI, IHDI, SDI, HAQ, population with at least some secondary education, life expectancy, and physicians per 10,000 people) had a significantly negative correlation with age-standardized DALY rate (all *p* < 0.001, Table 1).

**Table 1:** Univariate linear regression analysis of the correlation between the burden of hypertensive heart disease and the socioeconomic variables

Variate	R suqare	*P* value	β	95%CI
HDI	0.37	< 0.001	− 1150.97	− 1405.59 to − 896.35
IHDI	0.35	< 0.001	− 938.13	− 1152.72 to − 723.54
Life expectancy	0.42	< 0.001	−25.19	−30.16 to − 20.22
Education	0.18	< 0.001	−4.30	−5.83 to −2.77
HAQ	0.300	< 0.001	−11.21	−14.11 to − 8.31
SDI	0.27	< 0.001	− 862.48	− 1099.95 to − 625.01
Physician number	0.17	< 0.001	−7.38	−10.08 to −4.68

*SDI* socio-demographic index, *HDI* human development index, *IHDI* inequality-adjusted HDI, *HAQ* health access and quality Index

## Discussion

In this study based on the GBD 2019, we comprehensively analyzed the global burden and trends of HHD over three decades. Our results indicated that the prevalence of HHD in 2019 was higher than that in 1990, which may be explained partly by the life expectancy improvement and the global population aging and growth [17]. The higher prevalence of hypertension due to the revised diagnosis criteria published in 2017 may be another reason for increased HHD prevalence [18]. Meanwhile, the ASPR was higher in East Asia, Oceania, Southeast Asia, and North Africa and Middle East; it was lower in Eastern Europe, Australasia, high-income Asia Pacific, and South Asia, which was consistent with the prevalence of hypertension [19]. Obesity, unhealthy diet, lack of physical activity and genetic factors may interpret partly these disparities of HHD prevalence across regions [20–22]. The age-standardized DALY rate of HHD in Central and Eastern Sub-Saharan Africa, Oceania, and Africa was consistently higher than that in other regions. Therefore, we should attach significance to the potential public health approaches behind the fast decline of the age-standardized DALY rate in high-income Asia Pacific, East Asia, Central Latin America, and Tropical Latin America.

Although there was a more remarkable downward trend of female DALY EAPC in comparison with that of male, the prevalence EAPC in men was lower than that in women. The Prevention of Renal and Vascular End-stage Disease (PREVEND) study demonstrated that HHD patients tended to be female, older, and have obesity [23], which indicate a possible reason for the higher female prevalence EAPC. Sex hormones may influence cardiac energy metabolism and cardiovascular biofunction, which have a vital role in the development of HHD [24]. For the population aged over 50, all ASRs of HHD increased dramatically in both 1990 and 2019. According to the World Health Organization report, the proportion of people aged over 60 will approximately double from 12 to 22% between 2015 and 2050 [25]. Therefore, the effective prevention and optimal treatment of HHD deserves more attention for older patients, governments and clinicians, especially in the countries with population aging, such as Japan, China and Italy.

In this study, a higher prevalence of HHD was more likely occurred in middle SDI countries, yet the higher mortality associated with HHD was skewed towards low-SDI countries. We also showed that there was a negative relationship between the burden of HHD and socioeconomic variables (HDI, IHDI, SDI, HAQ, population with at least some secondary education, life expectancy and physicians per 10,000 people).

It may be caused by poor governance, insufficient health care cost, and inefficient care delivery system, suggesting that a decline in the disease burden of HHD depends on society, family, clinicians, and individuals. The governments of low-SDI countries spend lower percentage of their gross domestic product on health systems than high-SDI countries (2% in low-SDI countries vs. 12% in high-SDI countries) [26]. Additionally, the limited health resources were divided into treatments of infectious disease, undernutrition, childhood diseases, and HHD. The low rate of disease awareness (25–38%) has led to higher death rates in the developing countries [27, 28]. Elevating public awareness of HHD worldwide could improve efficiently the current effect of screening, prevention, and treatment. Due to higher mortality in low-SDI countries, a comprehensive strategy based on health, education, and income should be implemented urgently in these countries to reduce the disease burden of HHD.

HHD is a disease associated strongly with a long-term increase of systemic blood pressure, which may progress to diastolic dysfunction, left ventricular hypertrophy, and heart failure with reduced ejection fraction [29]. Many previous studies have indicated that intensive blood pressure control could improve the left ventricular ejection fraction and reverse cardiac remodeling [30, 31]. Some high-risk patients could benefit from lower systolic blood pressure (less than 130 mmHg) [32]. Therefore, with wide application of the revised criteria for diagnosing hypertension published in 2017, intensive blood pressure control will significantly improve the prognosis of HHD patients. Moreover, smoking, diabetes mellitus, and obesity are major modifiable risk factors for HHD [33]. Therapeutic lifestyle changes, including smoke cessation, low-salt and fat intake, and proper exercise, should be addressed for decreasing the HHD disease burden [34].

Our study had a few limitations. First, the actual data of HHD are not available in the GBD 2019 study, albeit GBD provides the high-quality estimations of disease burden. Second, there was an absence of detailed information on the clinicopathological features of HHD in the GBD 2019 study, including severity stage and laboratory examination. Third, we did not perform multiple linear regression in this study due to the collinearity of the available socioeconomic factors and lack of other relevant covariates. Despite these limitations, this analysis provided a comprehensive and up-to-date review of HHD disease burden at global, regional, and national levels.

## Conclusions

HHD is a public health issue around the world. The prevalence has been rising continuously during 1990–2019, even though the ASDR has decreased. HHD occurred mostly in people aged over 65. Multi-faceted improvements associated with health, education, and finance could help to alleviate the disease burden of HHD. According to the current study, policymakers should make more efficient and effective public health policies in order to set out the early detection and management strategies for HHD.

## Supplementary Information

**Additional file 1: Figure S1.** The age-standardized prevalence (A), death (B), and DALY (C) cases for hypertensive heart disease by different continents, 1990-2019.

**Additional file 2: Figure S2.** The top 20 countries with high disease burden (A, number of prevalence; B, prevalence rates; C, age-standardized prevalence rates; D, number of death; E, death rates; F, age-standardized death rates).

**Additional file 3: Table S1.** The global and regional burden of hypertensive heart disease.

**Additional file 4: Table S2.** The age-standardized rate of hypertensive heart disease in different SDI regions.

**Additional file 5: Table S3.** The global and regional age-standardized variation trends of hypertensive heart disease from 1990 to 2019.

**Additional file 6: Table S4.** The prevalence, death and DALYs of hypertensive heart disease in different regions.

**Additional file 7: Table S5.** The age-standardized rate of hypertensive heart disease in different regions.

## Data Availability

Publicly available datasets were analyzed in this study. The data can be found here: http://ghdx.healthdata.org/gbd-results-tool and http://hdr.undp.org/en/data.
