# Supplementary figures and images for: Global, regional, and national burden of hypertensive heart disease during 1990–2019: an analysis of the global burden of disease study 2019
Source: BMC Public Health. 2022 Apr 27;22:841. doi: 10.1186/s12889-022-13271-0 (PMC9044894; doi:10.1186/s12889-022-13271-0)

**A**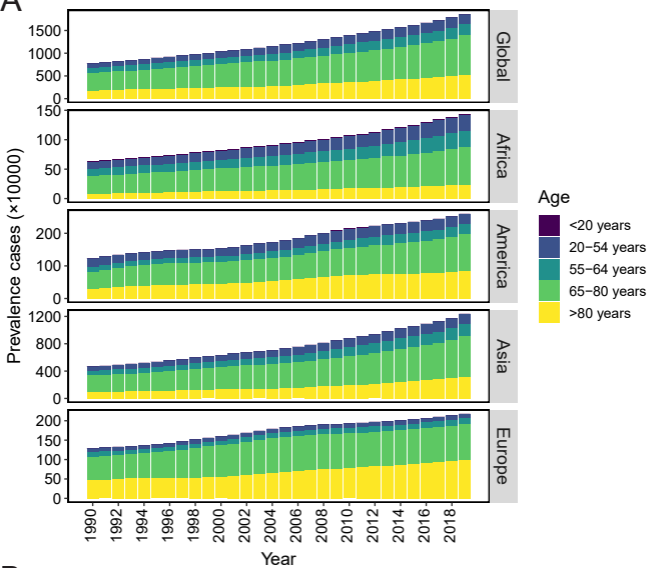**B**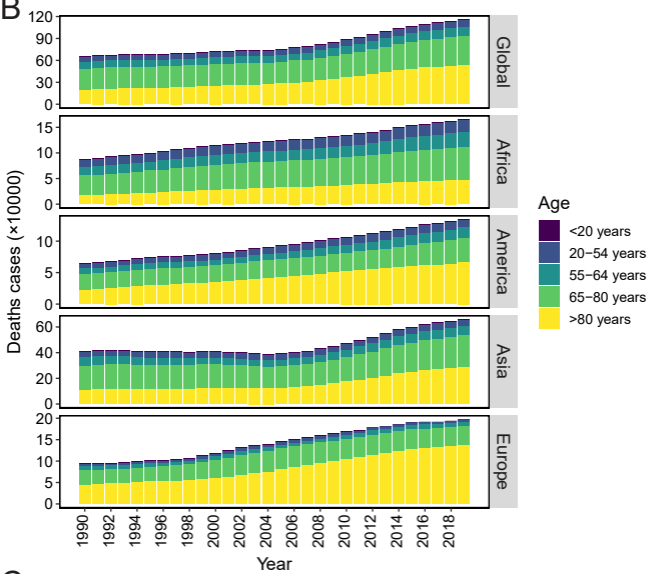**C**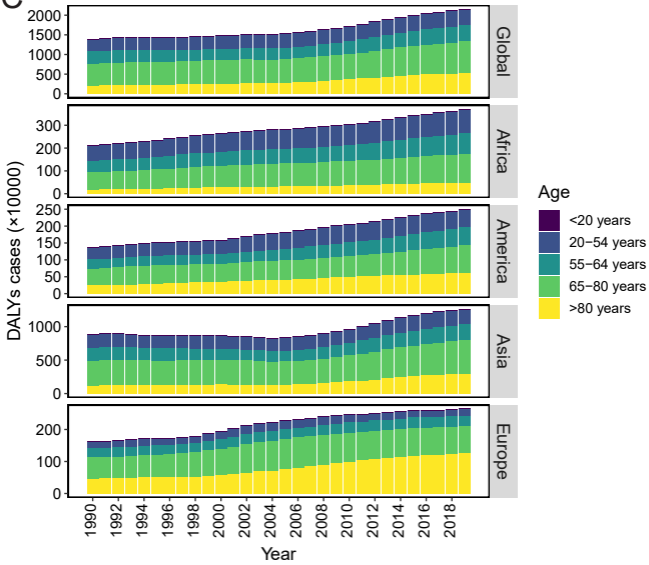

Supplement: Supplementary file 1 — Additional file 1: Figure S1. The age-standardized prevalence (A), death (B), and DALY (C) cases for hypertensive heart disease by different continents, 1990-2019. [file 12889_2022_13271_MOESM1_ESM.pdf]

A

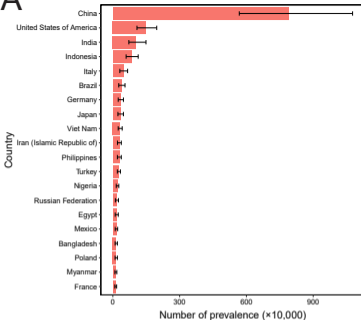

B

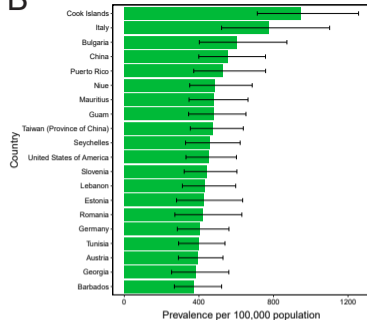

C

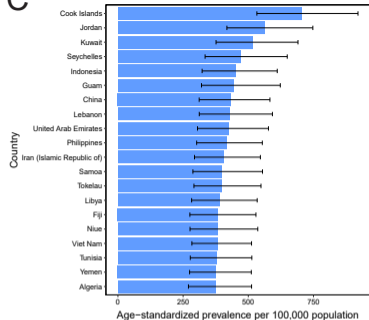

D

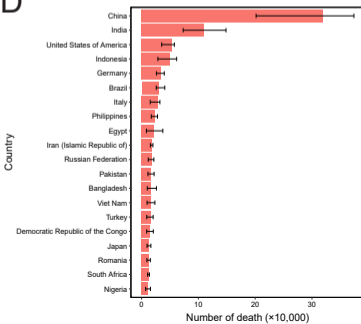

E

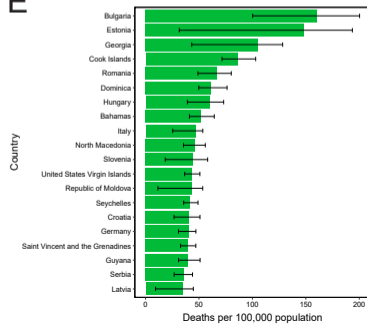

F

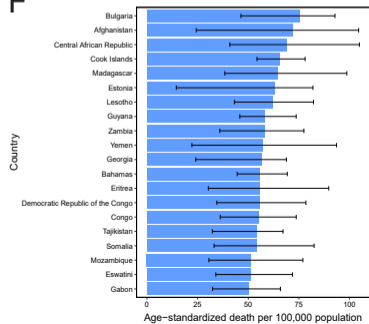

Supplement: Supplementary file 2 — Additional file 2: Figure S2. The top 20 countries with high disease burden (A, number of prevalence; B, prevalence rates; C, age-standardized prevalence rates; D, number of death; E, death rates; F, age-standardized death rates). [file 12889_2022_13271_MOESM2_ESM.pdf]
